# Endometriosis at Cesarean Scar Presenting As an Acute Abdomen

**DOI:** 10.7759/cureus.7350

**Published:** 2020-03-21

**Authors:** Nayana Gaba, Saurabh Gaba, Monica Gupta, Mandeep Singla, Ashish Dua

**Affiliations:** 1 Obstetrics and Gynaecology, Postgraduate Institute of Medical Education and Research, Chandigarh, IND; 2 Internal Medicine, Government Medical College and Hospital, Chandigarh, IND; 3 Radiodiagnosis, Government Medical College and Hospital, Chandigarh, IND

**Keywords:** scar endometriosis, acute abdomen

## Abstract

A 30-year-old female presented to the emergency department with severe abdominal pain and vomitings during her premenstrual period. Abdominal imaging revealed a mass originating from the scar of cesarean section extending into the rectus muscle. The cesarean section was performed four years back. Her history was significant for pain at the site of lesion during menses associated with disproportionate nausea. She was managed conservatively and improved. Fine needle aspiration cytology of the lesion established the diagnosis of scar endometriosis, and there was a permanent resolution of symptoms after its resection.

## Introduction

Endometriosis is the benign growth of endometrial tissue at sites other than the uterus. These ectopic sites are usually the ovaries, fallopian tubes, and uterosacral ligaments. Intestines, urinary bladder, cervix, and surgical scar sites are seldom implicated [1]. Principal symptoms include lower abdominal pain, infertility, and dyspareunia. The pain is generally cyclical and associated with menses, although some patients can have chronic pain. Here we are reporting the case of a 30-year-old female with scar endometriosis, which was diagnosed after she presented with an acute abdomen.

## Case presentation

The patient had undergone a lower segment cesarean section through a vertical midline incision four years earlier in view of fetal distress. It was her first pregnancy, and the baby was healthy. Her menses resumed three months after delivery. There was a progressive increase in pain associated with menses over the next few months and it was considerably more than what she used to experience before delivery. It was accompanied by excessive nausea and pain at the scar site.

She presented to casualty with severe abdominal pain and intractable vomitings during the premenstrual period. The pain was localized to the anterior pelvic area and was non-colicky. There was no history of abdominal trauma, diarrhea, constipation, or dysuria. She was not able to take anything orally. On examination, she was dehydrated and afebrile with a pulse of 110 bpm and a blood pressure of 100/70 mm Hg. There was tenderness over the scar, and a mass could be palpated below it. The abdomen was not distended. Bowel sounds were present, and she was passing flatus. Oral feeding was halted, and she was managed with intravenous fluids, analgesics, and empirical ceftriaxone and metronidazole.

**Figure 1:**
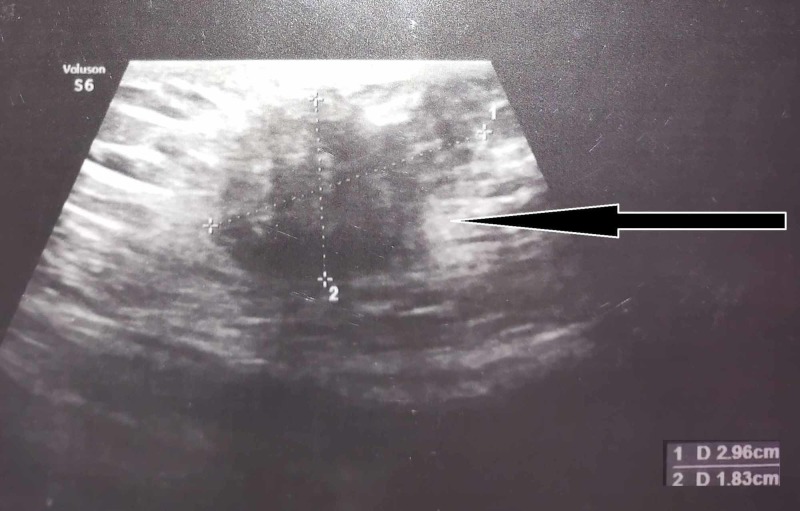
Ultrasound of the abdomen showing a hypoechoic mass (endometrioma) below the cesarean scar.

Preliminary blood investigations revealed normal blood count, and renal and hepatic functions. Serum electrolytes, amylase, and lipase were also within the normal range. The abdominal X-ray did not show any abnormality. Ultrasound (Figure 1) of the abdomen reported a relatively well-defined, slightly irregular hypoechoic lesion of size 2.96 x 1.83 cm in the anterior abdominal wall along the cesarean scar. The spiculated margins infiltrated the sheath of the rectus abdominis, and the mass had mild internal vascularity. Possibilities encompassed scar endometriosis and suture site granuloma. Rest of the abdomen was described as normal.

**Figure 2:**
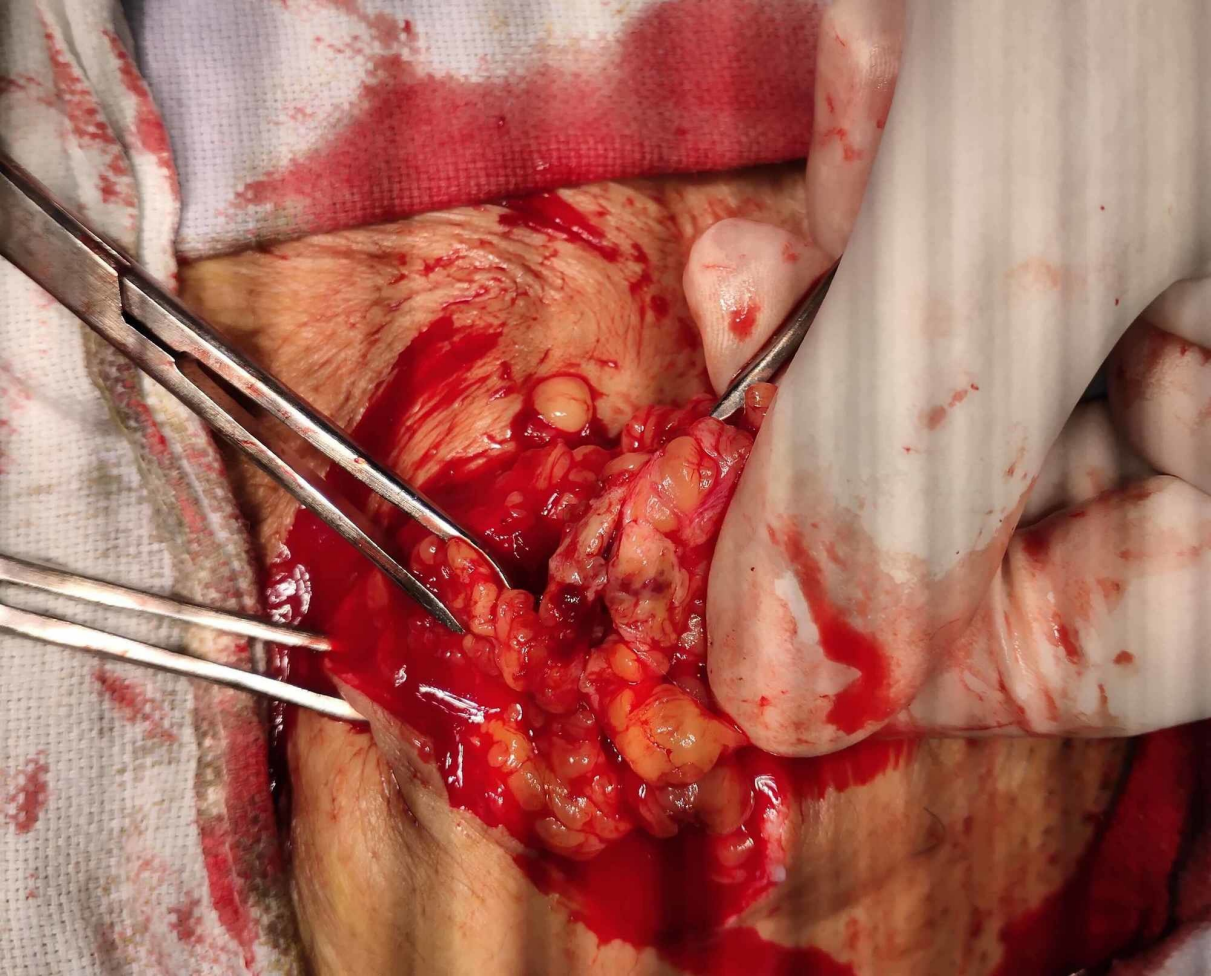
Endometrioma being excised.

**Figure 3:**
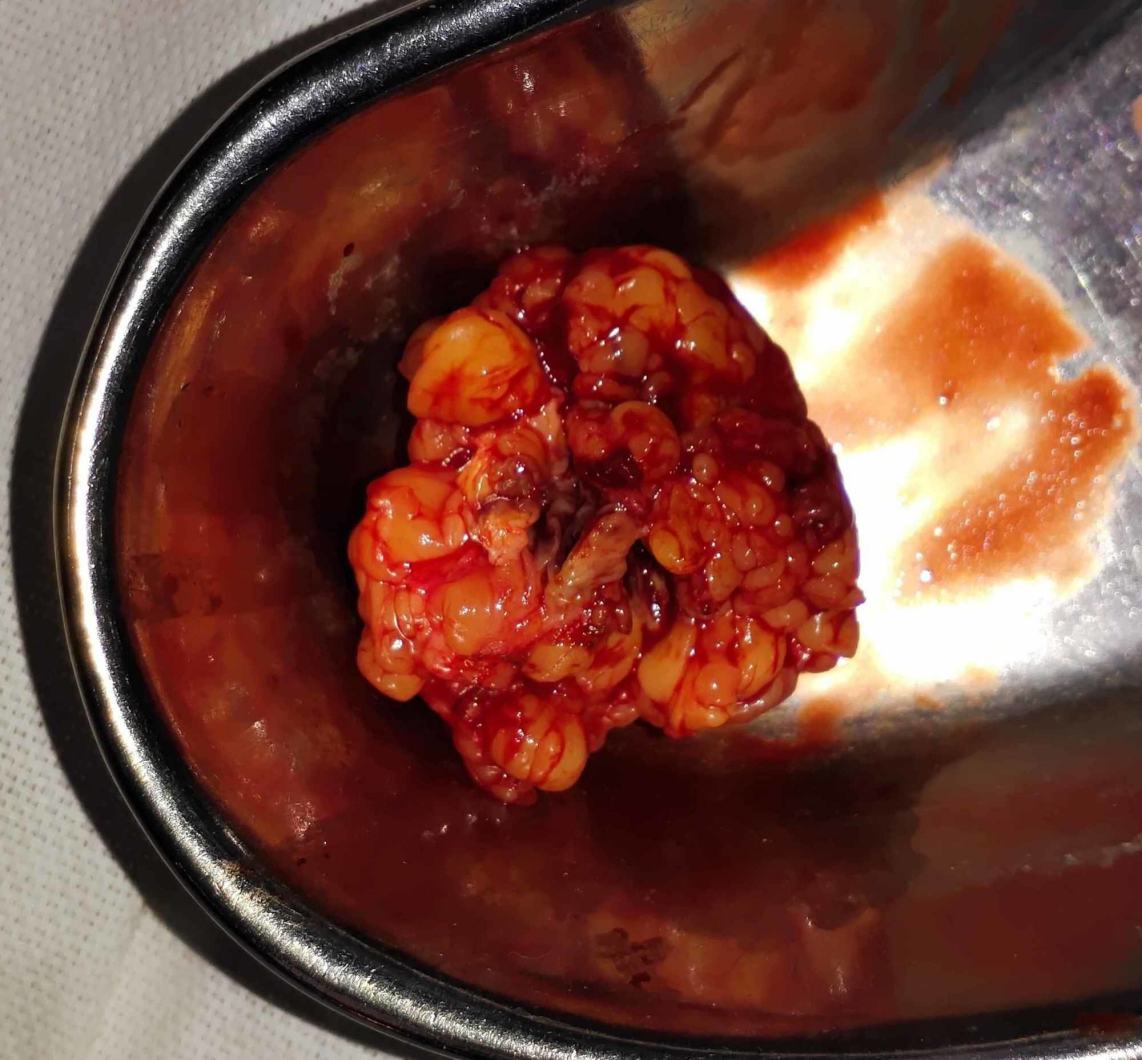
The excised endometrioma and accompanying adipose tissue.

Conservative management was continued, and, eventually, the patient recovered. She was able to take normal meals within three days. A fine needle aspiration biopsy from the mass was performed, and she was discharged. The biopsy revealed clusters and singly scattered endometrial cells with mild atypia along with stromal fragments, hemosiderin-laden macrophages, and scattered fibroblasts in the background. These features were consistent with scar endometriosis. The ectopic endometrial tissue was electively excised (Figures 2, 3), and the patient was relieved of all the symptoms.

## Discussion

The prevalence of endometriosis in females of reproductive age has been estimated to be 10-15% [2]. Around 25% of the patients are asymptomatic [1]. Pain is the predominant symptom and can be cyclical or constant, and the severity is not related to the extent or stage of disease [3]. The ectopic endometrial tissue can undergo cyclical morphological changes similar to normal endometrium during the menstrual cycle in response to hormone stimulation. Bleeding may also follow, which can elicit local inflammatory reaction. The dysmenorrhea is less common in ovarian endometrioses as compared with other locations [4]. The adverse social and psychological impact on the patients has been well documented [5]. Symptoms can also be non-specific and lead to diagnostic delay or attribution to other pathologies such as interstitial cystitis, pelvic inflammatory disease, and irritable bowel syndrome [5].

Etiology is unknown, but certain risk factors have been identified, such as low body mass index, nulliparity, early menarche, late menopause, and the presence of endometriosis in a first-degree relative [6]. The use of oral contraceptive pills has been shown to be protective [7]. Biopsy is essential for diagnosis, and the site is identified by imaging or direct visualization. Treatment modalities include the use of non-steroidal anti-inflammatory drugs (NSAIDS) to manage pain and hormonal treatment in the form of oral contraceptive pills and progestogen containing intrauterine device to suppress menstruation when pregnancy is not desired. Gonadotropin-releasing hormone agonists can be used for pain when pregnancy is desired. Surgical management is indicated when symptoms are severe or inadequately controlled by medical therapy and can be in the form of excision or ablation depending on the location and extent. Recurrence after surgical removal is to the tune of 50-60% after seven years [8]. There is some evidence to suggest that operating during the follicular phase reduces the recurrence and development of adhesions [9]. Around 20% of women do not have symptomatic improvement after surgery [10]. Furthermore, some patients may have a recurrence of pain without evidence of new lesions [11]. In a review of patients with endometriosis at the cesarean scar, it was found that the symptoms appeared after a mean period of 31.6 ± 23.9 months and it took another 28.3 ± 25.0 months for patients to be operated upon. The most common presentations were with abdominal mass and cyclical pain [12]. The patient in our report did not notice the mass below the scar, and the diagnosis would have been delayed had she not sought medical care during this episode.

## Conclusions

Endometriosis can have a spectrum of clinical manifestations, ranging from asymptomatic to severe abdominal pain requiring emergency visit, as in this case. The patient presented with an exaggerated premenstrual symptomatology of lower abdominal pain and vomitings, which left her dehydrated and incapable of taking food, raising concern of a sinister surgical pathology. However, the fact that no other abnormality was seen on the abdominal radiograph and ultrasound, along with swift improvement with conservative management alone, helped negate the need for a laparotomy. Histopathological examination of the mass arising from the cesarean scar confirmed the diagnosis of endometriosis and was later excised. There was no recurrence of symptoms. It is prudent for physicians and general surgeons to be mindful of the condition since they may infrequently encounter such patients. Taking the menstrual history can be of assistance, and the cyclical nature of symptoms is significant. Diagnosis is clinched by histological examination of the suspected lesion, and treatment depends on the symptoms.
